# Implementing Morpholino-Based Nucleic Acid Sensing on a Portable Surface Plasmon Resonance Instrument for Future Application in Environmental Monitoring

**DOI:** 10.3390/s18103259

**Published:** 2018-09-28

**Authors:** Andrea Bagi, Scott D. Soelberg, Clement E. Furlong, Thierry Baussant

**Affiliations:** 1 International Research Institute of Stavanger, 4068 Stavanger, Norway; Thierry.Baussant@norceresearch.no; 2 Departments of Medicine-Division of Medical Genetics and Genome Sciences, University of Washington, Seattle, WA 98195-5852, USA; scottjs@uw.edu (S.D.S.); clem@u.washington.edu (C.E.F.)

**Keywords:** surface plasmon resonance, 16S rRNA, bacterial detection, hydrocarbon biodegradation, magnetic bead based biosensing, portable biosensor

## Abstract

A portable surface plasmon resonance (SPR) instrument was tested for the first time for the detection of oligonucleotide sequences derived from the 16S rRNA gene of *Oleispira antarctica* RB-8, a bioindicator species of marine oil contamination, using morpholino-functionalized sensor surfaces. We evaluated the stability and specificity of morpholino coated sensor surfaces and tested two signal amplification regimes: (1) sequential injection of sample followed by magnetic bead amplifier and (2) a single injection of magnetic bead captured oligo. We found that the sensor surfaces could be regenerated for at least 85 consecutive sample injections without significant loss of signal intensity. Regarding specificity, the assay clearly differentiated analytes with only one or two mismatches. Signal intensities of mismatch oligos were lower than the exact match target at identical concentrations down to 200 nM, in standard phosphate buffered saline with 0.1 % Tween-20 added. Signal amplification was achieved with both strategies; however, significantly higher response was observed with the sequential approach (up to 16-fold), where first the binding of biotin-probe-labeled target oligo took place on the sensor surface, followed by the binding of the streptavidin magnetic beads onto the immobilized targets. Our experiments so far indicate that a simple coating procedure in combination with a relatively cost-efficient magnetic-bead-based signal amplification will provide robust SPR based nucleic acid sensing down to 0.5 nM of a 45-nucleotide long oligo target (7.2 ng/mL).

## 1. Introduction

Molecular techniques in general, and more specifically, nucleic acid sensing techniques are gradually replacing standard cultivation-dependent laboratory methods in both the public health sector, and environmental monitoring [1,2,3,4,5]. Biosensors in particular are very promising due to their versatility and suitability for miniaturization and integration in autonomous devices [6,7,8]. A number of signal transduction mechanisms exist and have been explored over the last decades for nucleic acid biosensing. Electrochemical sensors are utilized in the majority of the studies reviewed recently by Du et al. [9]. Surface plasmon resonance (SPR) is a relatively young approach in nucleic acid biosensing, nevertheless it is becoming a popular tool for label-free sensing of a variety of analytes [10,11]. To mention a few nucleic acid sensing examples, SPR has been applied for bacterial 16S rRNA, single-nucleotide polymorphism and microRNA detection [12,13,14]. SPR sensing is also currently being implemented on the third generation environmental sample processor (3G-ESP), a device capable of fully autonomous sample collection, analysis, and data transmission over extended deployment periods in the pelagic marine environment [15]. More specifically, the custom-made portable SPR instrument used in this study [16] has been integrated into the 3G-ESP recently. In situ ocean observing systems, such as the ESP have long been recognized as promising tools to provide real-time and high-resolution spatiotemporal data for risk assessment and monitoring of marine environments. Tools like ESP are needed especially in the new frontier of future oil and gas exploration and production activities, i.e., the Arctic and deep waters, to address the problems associated with traditional monitoring approaches [17,18,19]. Autonomous devices equipped with molecular tools can rapidly detect changes in environmental indicators, including bacterial species that are well-known to respond quickly to oil discharges. Among the obligate hydrocarbon (HC) degraders, the genus Oleispira is one of the most commonly reported cold-adapted alkane degrading genus. It has been identified both in deep-water sediments and the pelagic zone of cold marine habitats. Its genome has been sequenced and biodegradation potential mapped using various HC sources [20,21]. Oleispira was also among the HC-degraders monitored by Królicka et al. who proposed the use of mainly obligate HC degrading bacterial indicator species for the detection of oil leaks [22]. Their study showed the potential application of qPCR quantification of *Oleispira antarctia* 16S rRNA gene copy number as an indicator of oil exposure, using DNA extraction and qPCR protocols mimicking 2G-ESP procedures. Nevertheless, qPCR is a rather complex and time-consuming technique in comparison to nucleic acid sensing, particularly label-free SPR. SPR-based nucleic acid sensing can be achieved using various recognition elements, including natural DNA oligonucleotides and artificial analogues, such as peptide nucleic acids (PNA), linked nucleic acids (LNA), and morpholinos [23]. These analogues have properties such as better stability on the sensor surface and higher binding strength in comparison to natural DNA probes. Morpholinos are of particular interest as they are extremely stable and show a number of other advantages over their natural DNA counterparts and the other synthetic analogues [24]. Morpholinos are mainly used for in vivo silencing and knock-out experiments due to their resistance to enzymatic degradation [25]. Nevertheless, they are becoming increasingly used in biosensing and biodistribution measurements [26]. The SPR instrument developed in 2007 [16] has so far been used for antibody-based assays, with the aim to detect small molecules, as well as viruses and whole bacteria. Its sensor chip can also be coated with other types of recognition molecules. In this study, we have explored morpholinos as recognition elements to be applied for nucleic acid sensing on this platform. We chose to work with synthetic oligonucleotides representing the 16S rRNA gene sequence of *Oleispira antarctica* RB-8 in order to develop an assay for future use in 16S rRNA based environmental monitoring of oil pollution events. Our goals were: (1) to test whether an extremely simple coating procedure using morpholinos as recognition elements can provide suitable and robust (regenerable) sensor surface, (2) to assess the specificity of the assay, and (3) to test magnetic-bead-based signal amplification strategies.

## 2. Materials and Methods

### 2.1. General Assay Procedures and Materials

#### 2.1.1. SPR Instrument

**Figure 1:**
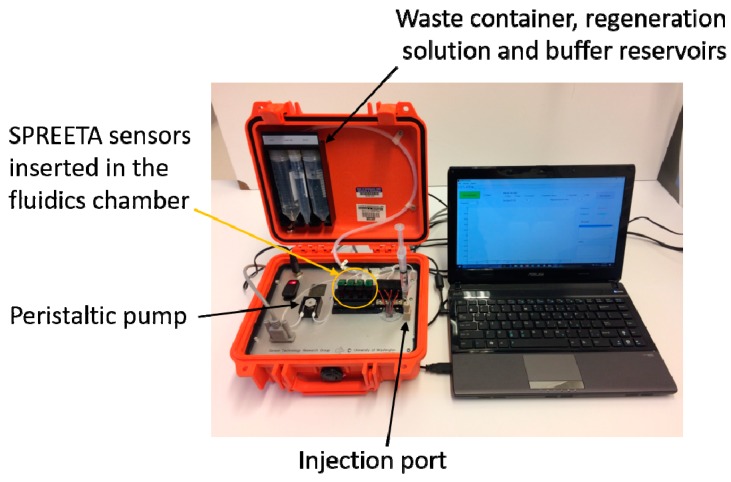
Photo of the portable surface plasmon resonance instrument connected to a 13” laptop.

All experiments were carried out with a 4-chip, 12-channel portable SPR instrument (Figure 1) manufactured at the University of Washington (Seattle, WA, USA). The current model is a modified version of the instrument described in [27]. The system is housed in a 27 × 25 × 12 cm case and incorporates miniature electronic and fluidic components. It includes temperature control and conditioning of the injected sample (≈±0.01 °C), which is a critical parameter of SPR use in field applications or for autonomous environmental sensor applications. The four-chip system is low power (2.6 watts average at 12 volts) and has low flow cell volume (1 µL per channel) and can be used as a 12-channel system. A computer graphical user interface (GUI) was also developed for data display and system control. The system was also designed to run semi-autonomously. Autonomous operation requires a ‘start’ signal be sent, followed by a sample being pumped or injected into the holding loop by the user or an outside sample processor. The system then processes the injected sample automatically through a series of low power/low dead-volume valves. Following a detection sequence, the system automatically performs a regeneration sequence by flowing a regeneration solution over the surface followed by flow of starting buffer to reset the system for another detection cycle.

#### 2.1.2. Probes

A 24-nucleotide hybridization probe previously used in a sandwich hybridization assay (personal communication Królicka, A.) was employed as the surface immobilized detection probe (DP). A morpholino oligo of this DP sequence with a disulfide-amide modification at the 3′ end was purchased from Gene Tools LLC (Philomath, OR, USA). The disulfide-amide modification at the 3′ end was used for attachment of the morpholino to the gold sensor surface. DP was checked against the Silva small subunit ribosomal RNA (16S SSU) database (Version 132, on 3 September 2019) using the online SILVA probe match and evaluation tool, TestProbe 3.0 [28]. Hits with 0 mismatches all belonged to the genus Oleispira, confirming the specificity of the DP probe. A previously published universal bacterial primer was used as the capture probe (CP) [29]. This was purchased in 5′-biotinylated DNA oligo form (IDT, San Jose, CA, USA). Sequences are shown in Table 1.

#### 2.1.3. Analytes

A 24-nucleotide-long segment of the Oleispira 16S rRNA gene (100% complementary to DP) was used as a reference sequence (REF) in all experiments, while a 45-nucleotide long combined oligo, complementary to both DP and CP was used as positive control (PC) in the amplification tests. PC contained an internal spacer (iSp18, an 18-mer hexa-ethylene-glycol spacer) between the segments complementary to DP and CP. For specificity testing, mismatched oligos containing 1 (M1) or 2 (M2) mismatches to DP were used. To test specificity, an oligo complementary to the universal CP was used as a negative control (NC, 15 mismatches to REF). Sequences are summarized in Table 1.

#### 2.1.4. Buffers

Dulbecco’s phosphate buffered saline (DPBS) purchased from Sigma-Aldrich Norway AS (Oslo, Norway) containing KCl (0.2 g/L), NaCl (8.0 g/L), KH_2_PO_4_ (0.2 g/L), anhydrous Na_2_HPO_4_ (1.15 g/L), and DPBS amended with 0.1% Tween-20 (DPBS-T) were used as hybridization and running buffer solutions, respectively. A 50 mM NaOH denaturing solution was used for regenerating the SPR surfaces.

#### 2.1.5. Preparation of Sensor Surfaces

Gold surfaces of the SPR sensors were cleaned with a piranha solution (Caution: piranha solution is extremely oxidizing and must not be stored in tightly capped containers on account of gas evolution) prepared by slowly adding 30% H_2_O_2_ to a concentrated sulfuric acid solution (three drops H_2_O_2_ added to nine drops H_2_SO_4_). Approximately 40 µL of this solution was used to cover the gold surface of the sensor for 20 min. The sensor surfaces were then washed with deionized water. Morpholino stock (1 mM in molecular grade water) was first diluted to 100 µM in DPBS, then a 20 µL droplet was carefully applied onto the clean gold surface of a sensor chip. Coated sensors were incubated at room temperature in a dark humid chamber at least overnight. Prior to use, the gold surface was rinsed with 1 mL DPBS-T, placed in the sensor slot and referenced with sucrose solution (30 w/v %). For storage, the functionalized surfaces were wicked dry and then stored in a plastic case under dark and dry conditions for up to two months.

#### 2.1.6. Experimental Procedures

Each analysis was performed using the fluidics program outlined in Table 2. Note that the baseline was established during the two-minute-long initialization step, then the sensors were ‘zeroed’ during the baseline step. The fluidics program was slightly modified for testing the sequential approach, where an additional injection step and subsequent flow and flush steps were included. The total duration of one measurement was between 12 and 24 min, with binding time kept constant at 6 min with a flush step after 4 min.

To monitor stability and potential degradation of the signal, a 500 µL aliquot of the reference oligo (REF) at 5 µM concentration was injected and analyzed at the beginning and at the end of each experiment. RIU values at 6 min were recorded and tracked over time. Similarly, the signal of buffer injections was also tracked.

#### 2.1.7. Preparation of Oligos and Magnetic Beads

All oligos were prepared in molecular grade water as 1 mM concentration stocks and stored at 4 °C. These were diluted in DPBS-T prior to injection and analysis unless otherwise stated. Streptavidin coated magnetic beads (500 nm diameter, 2 mg/mL, BD IMag™ Streptavidin Particles Plus-DM, catalog no. 557812, BD Biosciences, Franklin Lakes, NJ, USA) were washed twice in DPBS and were resuspended in DPBS, in a volume equal to that taken from the original stock. This solution is subsequently called: 1× beads.

### 2.2. Testing Specificity

The reference sequence (REF), two mismatch oligos (M1 and M2), and a negative control (NC) oligo were injected at various concentrations (50–5000 nM) and RIU values recorded after 6 min of binding were compared. A buffer injection was always carried out between different samples and the RIU values of these injections were used as background values. Oligos were diluted in DPBS-T to final concentrations of 50, 200, 1000, or 5000 nM. Measurements were carried out in triplicate except for the 5000 nM samples. Pairwise comparisons between the signal of different oligos at a given concentration were carried out with JMP 5.1 software using Student’s *t*-test (*α* = 0.05).

### 2.3. Testing Two Signal Amplification Strategies

#### 2.3.1. Sequential Assay

**Figure 2:**
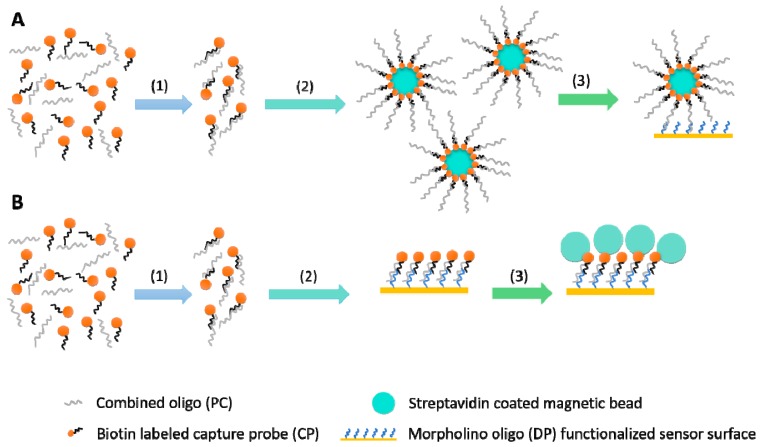
Illustration of the two different amplification strategies: capture approach (**A**) and sequential approach (**B**). The first step (1) was identical in both cases: the hybridization of positive control (PC) oligo with the biotin labeled capture probe (CP) took place. The second step (2) in the capture approach was to conjugate the biotin labeled hybridized oligo-probe complex to the streptavidin coated magnetic beads, while in the sequential approach, the oligo-probe complex was injected and its binding to the morpholino functionalized surface was recorded. Finally, in the third step (3) of the capture approach, oligo-probe complex conjugated magnetic beads were injected and a single binding curve was recorded. In case of the sequential approach, streptavidin coated magnetic beads were injected and the second binding curve, binding of beads to immobilized biotin-labeled oligo-probe complex was recorded.

PC oligo and biotin labeled CP probe stock solutions (1 mM) were diluted 10-fold in DPBS buffer. Then 2.5 µL of diluted PC (100 µM) and 2.5 µL of diluted CP (100 µM) were mixed and allowed to hybridize for 20 min at room temperature (approx. 20 °C). DPBS-T buffer (495 µL) was then added and the hybridized oligo and probe complex was injected. The binding curve was recorded for 6 min then 1× beads (5, 10, 25, or 50 µL = 10, 20, 50, and 100 µg, respectively) resuspended in DPBS-T (final volume of 500 µL) were injected for the amplification step (Figure 2B). Refractive index units (RIU) values were recorded after 6 min. Measurements were carried out in triplicates. The signal of the bead solution alone was also recorded in triplicate at the beginning of the experiment for each bead concentration as background and subtracted from the sample signals.

#### 2.3.2. Capture Assay

PC oligo and biotin labeled CP probe stock solutions (1 mM) were diluted 10-fold in DPBS buffer, then 2.5 µL diluted PC (100 µM) and 2.5 µL diluted CP (100 µM) was mixed and allowed to hybridize for 20 min at room temperature (approx. 20 °C). Afterwards, DPBS buffer (445–490 µL depending on the volume of streptavidin beads) and 1× beads (5, 10, 25, or 50 µL = 10, 20, 50, and 100 µg, respectively) were added and mixed. Following a 10 min incubation at room temperature, the mix was placed on a magnetic rack and separation was carried out for at least 10 min. Supernatant was then removed with a pipette, conjugated beads were resuspended in 500 µL DPBS-T running buffer and injected immediately (Figure 2A). RIU values were recorded after 6 min. Measurements were carried out in triplicates.

### 2.4. Testing the Detection Limits

Detection limits were tested using the REF oligo alone and using the PC oligo in combination with sequential magnetic bead signal amplification. For the first experiment, a serial dilution of the REF oligo samples at concentrations of 50, 200, 500, 1000, and 5000 nM were analyzed using the same fluidics program described in Table 2. For the second experiment, a 10-fold serial dilution of the PC oligo (0.1–100 µM) was prepared and assays were carried out as described for the sequential approach with final concentrations of PC at 0.5, 5, 50, and 500 nM. The concentration of CP probe working solution was kept constant at 100 µM for all tested PC concentrations to ensure excess. All measurements were carried out in triplicates.

## 3. Results and Discussion

### 3.1. General Assay Procedures

**Figure 3:**
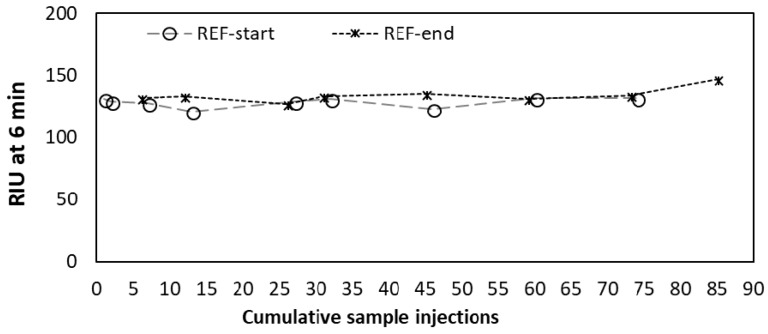
Refractive index unit (RIU) measured after 6 min of incubation for the reference sequence (REF) at the beginning (REF-start) and at the end (REF-end) of each experiment.

Preparation of SPR sensor surfaces can require elaborate procedures (largely depending on the chemistry used for binding the oligo to the gold surface) in order to ensure optimal coverage, probe orientation, and to minimize the surface area of bare-gold sites available for non-specific adsorption [30]. This is however not always necessary, instead rather simple coating procedures can be sufficient to improve the orientation of the DNA probes and to attenuate non-specific binding, such as a short post-treatment with 6-mercapto-1-hexanol [12] or using 11-mercaptoundecanoic acid as a linker for immobilizing ssDNA probes [31]. Surface passivation of thiol-modified morpholino coated sensors can be performed similarly by applying short alkanethiols, but this process requires careful optimization of alkanethiol concentration and incubation time [32]. Our approach was even simpler, similar to the strategy reported by Liu et al. [33]. After cleaning the surfaces with piranha solution, the morpholino layer was created by incubating the surface with a droplet (20 µL) of concentrated (100 µM) morpholino probe solution overnight. The disulfide-amide modification used here, in contrast to the more widely used thiol (–SH) modification, enabled a strong and long-lasting bond between the gold surface and the morpholino probes. Measurement results of the REF oligo were nearly constant for over 85 sample injections (approx. 100 regeneration cycles) with no significant decrease in signal intensity as shown in Figure 3. This ability of maintaining responsivity (reusability) for such a large number of samples (i.e., regeneration cycles) is extremely advantageous when considering potential long-term deployment of automated remote sensing equipment.

**Figure 4:**
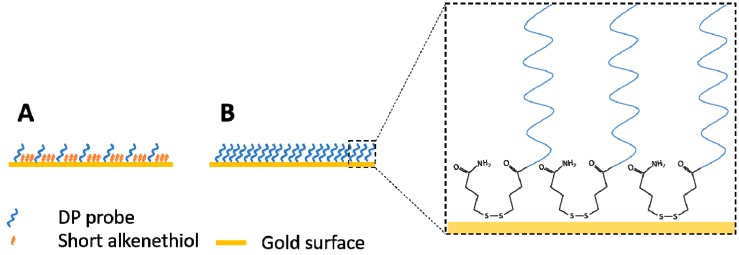
Illustration of the potential difference between a short alkanethiol passivated gold surface with lower probe coverage (**A**) and a high-density probe coverage without passivation step such as the case in this study (**B**). Chemical structure of the disulfide-amide modification at the 3′-end of the morpholino oligos is shown in the inset. DP probe = detection probe.

A possible reason for such a robust surface, besides the nature of sulfhydryl linkage, might be due to the high probe coverage of the sensor (Figure 4). The gold surface area on the SPREETA chip is approximately 4.5 × 9.5 mm (0.4275 cm^2^). During the coating procedure ~10^21^ molecules are introduced onto this area. Theoretically, this could result is a coverage of 2.8 × 10^21^ molecules/cm^2^ if all molecules could form a sulfhydryl linkage with the gold surface, however this is unlikely. A maximum coverage can be estimated assuming that the diameter of the morpholino probe is similar to ssDNA (~0.5 nm) [34], hence the area occupied by a single probe would be 0.785 nm^2^ leading to an estimate for the maximum number of molecules (of the DP probe) to attach to be ~5.4 × 10^13^. This is much closer to the coverage reported for a morpholino based biosensor (on the order of magnitude ~10^12^ molecules/cm^2^) [35]. The coverage of the sensors used here was most likely higher than the minimum coverage established for a surface plasmon diffraction sensor (~1.1 × 10^11^ molecules/cm^2^) [36]. For comparison, an optimal morpholino monolayer was prepared by incubation with 0.25 µM solution (in deionized water) resulting in 5 × 10^12^ probes/cm^2^ for an electrochemical sensor [32] while a self-assembled DNA oligo layer was prepared by incubating 10 μL of a 10 ng/µL (1 µM) concentration oligo solution on a gold surface by [33]. The large excess of morpholino introduced to the gold surface used in this study, most likely resulted in maximal coverage, ensuring high enough density of the morpholino probes on the SPR chip, leaving no bare-gold sites for non-specific adsorption (Figure 4). This may have also been the reason for the observed low levels of non-specific adsorption on morpholino coated sensors in comparison to bare-gold surfaces used as control (data not shown).

### 3.2. Testing Specificity

**Figure 5:**
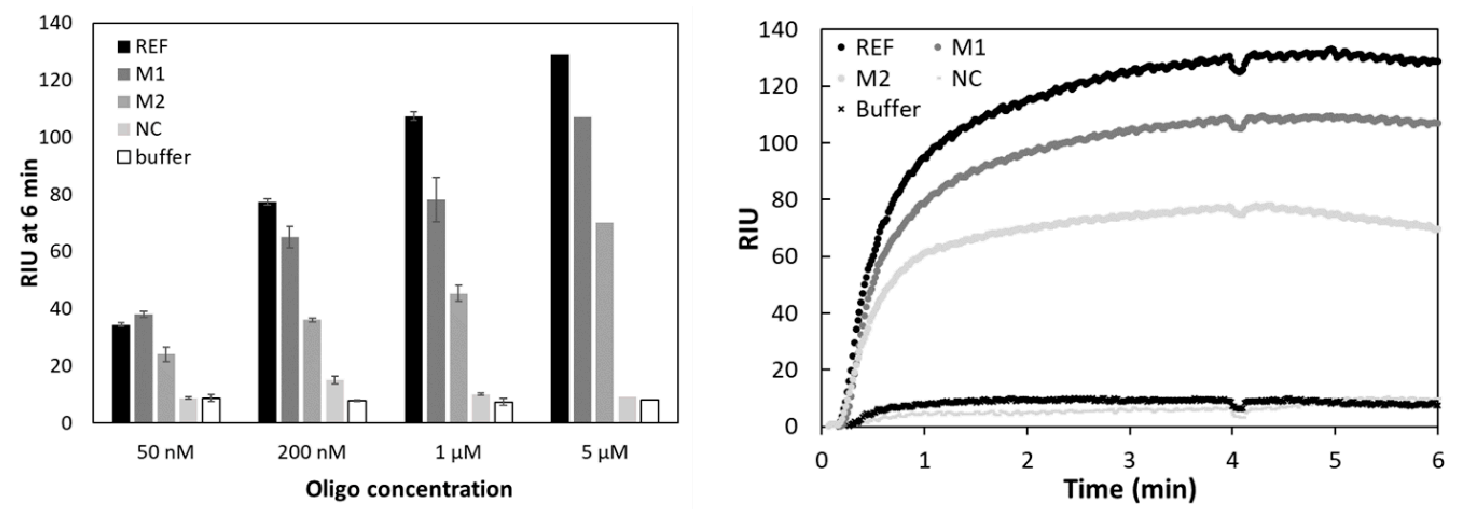
Refractive index units (RIU) measured after 6 min (**Left** panel) and binding curves at 5 µM concentration (**Right** panel) for 100% complementary (REF), one-mismatch (M1), two-mismatch (M2) and non-complementary (NC) sequences at four different concentrations. n = 3 except for the 5 µM case where only one set of measurements were carried out.

Morpholino probe-based biosensors are expected to exhibit superior selectivity over DNA oligo-based counterparts as mismatches have a higher destabilizing effect on morpholino-DNA hybrids than on DNA–DNA hybrids [23]. Morpholino-functionalized electrochemical biosensors in particular have reported high specificity discriminating one mismatch target from 100% complementary targets [29,36]. Observed lower affinity of morpholino probes, in comparison the DNA probes, was hypothesized to be responsible for enhanced selectivity–since longer perfect match regions are necessary for a high-enough-affinity binding between morpholino probe and its DNA target [37]. Moreover, morpholino-DNA hybrids can be formed at such low salt concentrations, which would normally prohibit DNA–DNA hybridization. While the binding affinity of morpholino probes towards DNA in solution is insensitive to the ionic strength of the buffer, surface immobilized morpholinos exhibit faster binding kinetics under high salt concentrations [23]. Signal intensity for the 100% complementary oligo (REF), 1 mismatch (M1), 2 mismatch (M2), and non-complementary (NC) sequences in this study, was recorded at four different oligo concentrations using a medium salt buffer (~150 mM NaCl in DPBS-T) (Figure 5, Left panel). As expected, the REF oligo gave the highest signal intensity, except for the lowest tested concentration (50 nM) where surprisingly the 1 mismatch containing oligo gave a higher signal, although the difference between REF and M1 was not significant (Student’s *t*-test, *p* > 0.05). The non-complementary sequence (containing 15 mismatches to DP) was nearly indistinguishable from the background (buffer injected), except for the 200 nM case where it was higher than the buffer signal, again, the difference was not significant (Student’s *t*-test, *p* > 0.05). DNA-probe based studies often reported similar results, in the sense that DNA or RNA analytes with only one or two mismatches give relatively high signals [38,39]. In contrast, a morpholino capture probe based electrochemical sensor for microRNA showed excellent selectivity at 100 fM miRNA concentration, with a signal of the one-mismatch target being 7% and a signal of the two-mismatch target being only 2% of the signal measured for the fully complementary sequence [40]. This was most likely achievable as a result of the temperature control employed, namely that the hybridization step took place at 60 °C ensuring specific annealing of probe to template. Such temperature control is usually not incorporated in SPR systems, which generally seem to struggle with mismatched targets containing only one or two mismatched oligonucleotides. Even a highly sensitive assay presented by Ding et al. [14] was characterized by a signal of the one-mismatch target being approx. 20% of the fully complementary sequence at 10 nM. This value dropped to approx. 16% at the 1 nM concentration level. Nevertheless, the signal intensity of the one- and two-mismatch targets at such low concentration (1 and 10 nM) was very close to that of the background while the 100% complementary target was significantly higher. It will be interesting to perform a mismatch test with an optimized amplification protocol and assess the selectivity of our assay at lower than 50 nM concentration. Finally, it is worth noting that one way proposed to distinguish mismatched oligos in case of DNA–DNA hybridization, was to compare the kinetics of the rinsing step, as the mismatched targets were apparently washed out faster [34]. This was however not observed in our case. The signal of M1 and M2 samples at 5 µM concentration did not show the large drop found by Yu et al. 2004 (Figure 5, right panel) [36].

Usually, the observations regarding mismatched targets are interpreted as the ability of the sensor to distinguish one- or two-mismatch oligos from the target. Under circumstances, when the analyte is extremely pure, and its concentration is known, such ability can be indeed used to discriminate a ‘mutant’ nucleic acid sequence from the ‘wild-type’ [13,41]. However, when it comes to specifically detecting a target biomarker species from an entire community of bacteria, this ‘ability’ can prove to be less advantageous. Detection of 16S rRNA genes from other non-oil degrading bacteria with one or two mismatches compared to the target species could lead to false-positive results, suggesting the presence of oil degrading bacteria when they are not present. Nevertheless, bacteria with only one or two mismatches in a given region of the 16S rRNA are likely phylogenetically closely related and may even be functionally similar to each other. This appears to be the case for some of the Oleispira-related marine hydrocarbon degrading bacteria. *Thalassolituus oleivorans* strain MIL-1, contains only a single mismatch in the DP-targeted region of its 16S rRNA in comparison to *Oleispira antarctica* RB-8, despite the overall sequence identity of 92% between the two strains. The 16S rRNA gene sequence of *Spongiispira norvegica* strain Gp_4_7.1, a closely related Oceanospirillacaea member had only two mismatches. Other closely related non-Oleispira strains reported to have similar physiology, *Bermanella marisrubri* strain RED65, and *Oceaniserpentilla haliotis* strain DSM 19503 had three and two mismatches, respectively. These observations illustrate that perhaps the approach reported here could be suitable to detect a suit of bacteria belonging to a functionally similar phylogenetic assemblage rather than a single species. Such a case was demonstrated by a chemiluminescence assay designed for marine Vibrio species, where the two selected Vibrio-specific probes (capture and signal) could differentiate 21 Vibrio vs. 10 non-Vibrio species at 20 ng/µL (~50 nM, M16Sr RNA = 491.528 kDa) total RNA concentration [42]. Using a dual Oleispira-specific probe (both capture and detection probe to be designed specifically for Oleispira) may be an alternative to increase specificity. In any case, assessment of hybridization-based assays as performed by Da-Silva et al. [42] are necessary and should be carried out to validate results with our SPR system as well.

### 3.3. Testing Two Signal Amplification Strategies

Magnetic beads (usually 1 µm diameter) are routinely used for nucleic acid separation procedures. In SPR systems, usually smaller diameter gold-nanoparticles are used for signal amplification [31,37,38]. However, they are more expensive and due to small size are amenable to colloidal aggregation. We chose to work with larger beads coated with a streptavidin monolayer as a more cost-efficient and a more stable alternative. Streptavidin alone was shown to be a promising signal amplifier [14]. Larger beads will also result in greater signal amplification as long as they can be captured by the evanescent wave. The sensor chip in the SPR system used in this study uses an 830 nm light-emitting diode light source, providing a probing distance of approximately 400 nm into the medium. Thus the 500 nm beads, which are polydisperse, ranging in size from 50–450 nm, with a mean of 200 nm are still within the range of detection of the SPREETA sensor. Another reason for focusing on using larger beads was that in the future, we plan to implement magnetic separation-based capture of bacterial rRNA prior to injection into the SPR system. This step can be carried out fastest by using larger diameter beads.

The two signal amplification strategies reported here were designed in accordance with the findings of Da-Silva et al. [39]. There the authors established that a prior hybridization of the biotin labeled capture probe with the target sequence followed by binding of this complex to the immobilized detection probe resulted in the highest response out of the three different strategies tested. Another possible approach would be to first conjugate the magnetic beads with the biotin labeled probes then perform the capture by hybridizing the target with the already conjugated probes (direct capture). However, indirect capture approach (hybridizing DNA-probe first then adding beads) seems better than direct capture (saturating beads with probes and then capturing by hybridization) according to [40]. Although the authors did not provide discussion regarding the possible reasons, this phenomenon may be explained in terms of relief of steric repulsion during binding of the oligonucleotides.

**Figure 6:**
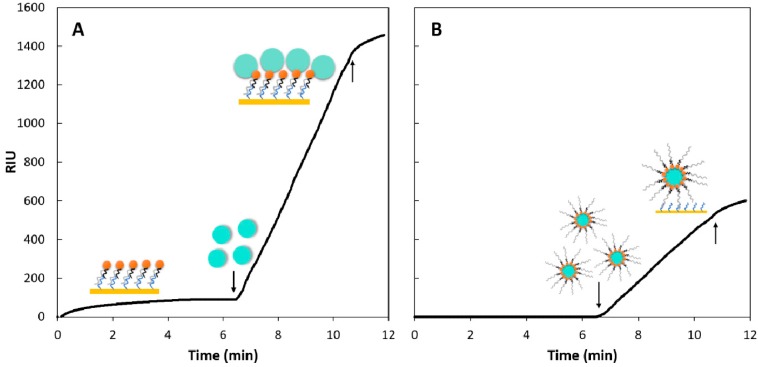
Refractive index units (RIU) recorded over time for the two amplification strategies, sequential (**A**) and capture (**B**) at identical target analyte (PC oligo) concentration (500 nM) and using the same amount of 1× beads (50 µL = 100 µg). The illustrations correspond to the steps described in the Materials and Methods section and symbols are identical to those used in Figure 2. The *y* axes were scaled to maximum 1600 RIU for both plots. The second, upward pointing arrow marks the ‘flush’ step, where the injection loop was flushed with buffer, hence no more analyte was being introduced from the injection loop to the sensor surface after this point.

In this study, we first tested whether 500 nm diameter beads resulted in significant signal amplification in our SPR system, then a capture approach was also evaluated. An example binding curve for the two strategies is shown in Figure 6. The first 6 min of the capture approach (Figure 6B) were set at constant 0 as there was nothing being injected at this stage while the oligo-probe complex was being injected in the sequential approach. The first downward facing arrow in Figure 6A points at the second injection step of the sequential approach, while in Figure 6B, it points at the first and only injection step of the capture approach. Both curves were recorded at 500 nM PC oligo concentration and 50 µL 1× bead amount.

**Figure 7:**
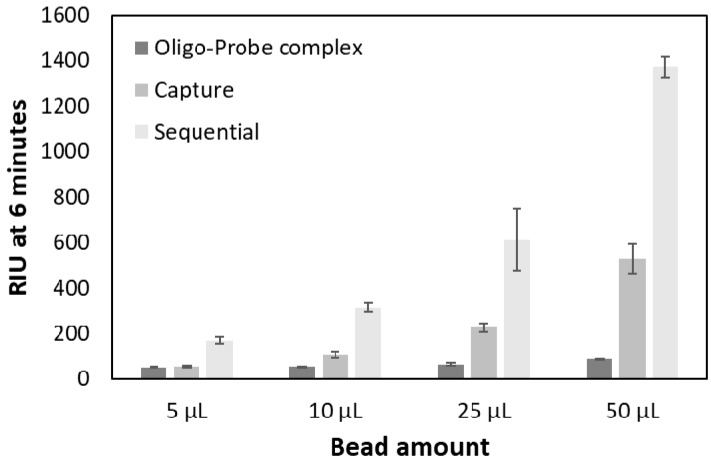
Comparison between the two signal amplification strategies at different bead amounts (5, 10, 25, and 50 µL) at constant oligo and probe concentration (500 nM).

The results showed that the magnetic beads indeed resulted in signal amplification (Figure 7) in comparison to injecting only the hybridized oligo-probe complex. Using 50 µL of the 1× bead stock solution (100 µg beads) resulted in the highest amplification factor for both strategies (Table 3).

**Figure 8:**
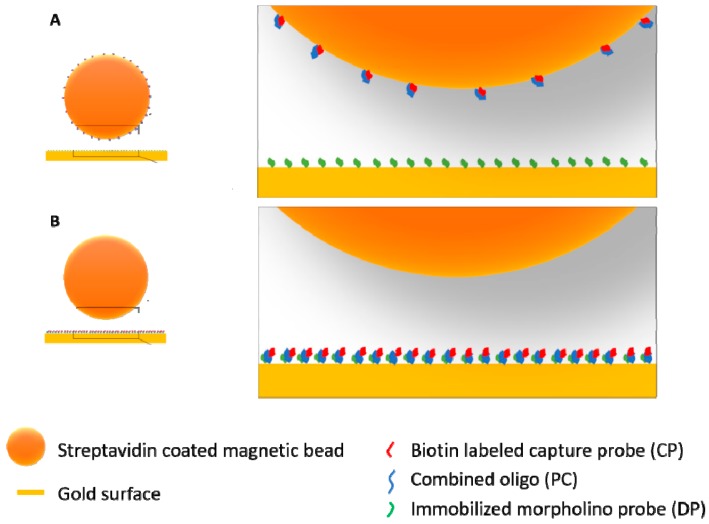
Illustration of the difference between the two signal amplification strategies, capture (**A**) and sequential (**B**) when it comes to the bead injection step. Oligo-probe conjugated beads are binding to the morpholino functionalized gold surface in the case of the capture approach (**A**) and bare-streptavidin coated beads are binding to the biotin labeled and immobilized oligo-probe complexes in the case of the sequential approach (**B**). The illustration is size-proportional, diameter of the magnetic particles is 500 nm while the length of DP and CP is ~8 nm while PC is ~17 nm long.

However, the signal observed with the sequential approach was significantly higher than that of the capture approach. After the capture experiment, the supernatant was collected and injected separately to confirm the presence of ‘leftover’ oligo-probe complexes. Such experiments showed that most likely there is a low efficiency in capturing the hybridized oligo-probe complexes onto the beads as there was clear binding taking place when injecting the supernatant (data not shown). This indicated the need for further optimizing the protocol for oligo capture in our future experiments. There could have been reasons, other than the capture procedure not being optimized yet, for the observed differences between the two strategies. To illustrate in a realistic manner the difference between the capture and the sequential approach, particularly in the bead injection step, a size-proportional image was created (Figure 8). This representation clearly shows that unless the coverage of the magnetic bead with conjugated oligo-probe complexes is as high as that of the gold surface, the probability of the bead binding to the surface seems to be reduced in the capture scenario (Figure 8A). It is also worth noticing that while in the sequential scenario, a biotin–streptavidin binding takes place, which is a fast and extremely high-affinity process (K_d_ on the order of ≈10^−14^ mol/L), in the case of the capture approach, a morpholino–DNA hybridization must occur in order for the bead to become bound. While morpholino–DNA hybridization is still a high affinity binding process, the dissociation constants (K_d_) of this reaction were found to be over 3 orders of magnitude higher, on the order of 10^−10^ mol/L (0.18 ± 0.02 nM for a 25-nucleotide tetramethyl rhodamine labeled morpholino probe and 0.31 ± 0.04 nM for a 25-nucleotide Alexa488 labeled morpholino probe) in a solution containing 10 mM Tris (pH 7.5) and 1 mM EDTA [41]. The same authors also determined hybridization rates of the morpholino probes to the target DNA in high salt (150 mM NaCl, similar to the DPBS-T buffer used in this study) and in low salt (5 mM NaCl) buffers and found that the rate was three times higher in the low salt ((15 ± 2) × 10^5^ M^−1^·s^−1^) in comparison to the high salt ((4.8 ± 0.5) × 10^5^ M^−1^·s^−1^). This suggests that lowering the salt concentration in our assay may improve the conditions for the binding of conjugated magnetic beads.

### 3.4. Testing the Detection Limits

**Figure 9:**
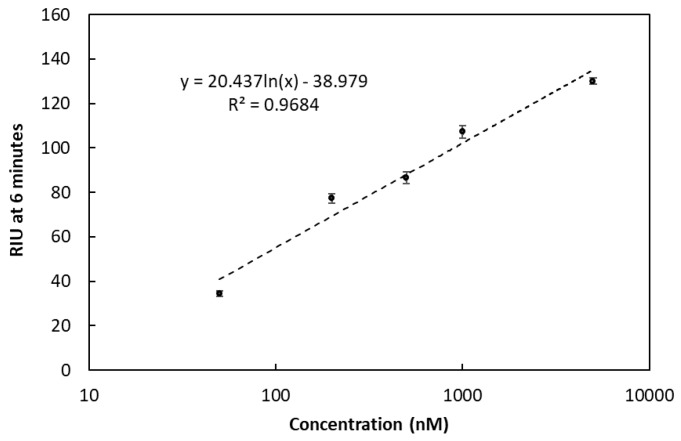
Refractive index units (RIU) at various concentrations (50, 200, 500, 1000, 5000 nM) of the Oleispira oligo (REF). For all measurement points *n* = 3. Error bars represent SD.

The average buffer signal over the 85 sample injections was 7.3 ± 2.5 RIU estimating an LOD at approximately 30 RIU (considering the limit to be at three times the background signal). Taking this into account, the lowest detectable concentration of the Oleispira oligo (REF) without any amplification was 50 nM (373 pg/µL). Results from the serial dilution of the REF oligo are shown in Figure 9.

**Figure 10:**
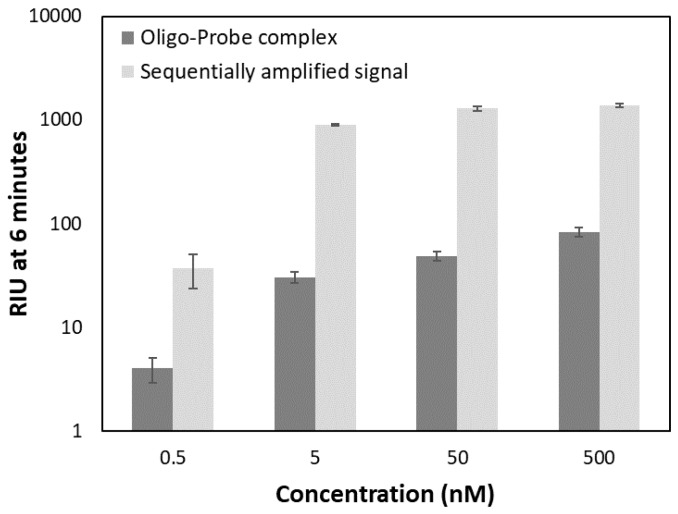
Concentration dependent response of the oligo-probe complex only and the signal of the same samples with sequential signal amplification approach using 50 µL 1× bead stock. Amplified signal refers to the refractive index units (RIU values) recorded at 6 min of binding once the beads were injected.

Measured RIU values for the 10-fold dilution series of the oligo-probe complex (0.5, 5, 50, 500 nM) using the sequential strategy at constant 50 µL 1× bead stock solution are summarized in Figure 10. A gradually decreasing signal was observed between 500–5 nM analyte concentration with a larger drop from 5 to 0.5 nM. While the signal of the oligo-probe complex alone at 0.5 nM concentration was below the 30 RIU established as the limit of detection, this level of the PC oligo was still detectable with magnetic bead-based signal amplification.

The SPR instrument used in this study has been employed for antibody-based detection of smaller (domoic acid, M = 311 g/mol and cortisol, M = 362 g/mol) and larger (staphylococcal enterotoxin B, SEB, M = 28,366 Da) analytes with detection limits reaching 10 nM (3.2 ng/mL) for domoic acid, 1 nM for cortisol (0.36 ng/mL), and 3.5 pM (0.1 ng/mL) for SEB, respectively [42,43,44]. Here we reported the first application of this portable SPR for oligonucleotide sensing, reaching detectable levels at 0.5 nM for the PC oligo (7.2 ng/mL) with the sequential amplification strategy. The lowest measured concentration here (0.5 nM) theoretically corresponds to 250 pg/µL of the ~1500 base long rRNA sequence of *Oleispira* (491.528 kDa), meaning that approximately 125 ng rRNA would be necessary to be injected (given the 500 µL injection volume) in an assay. This is approximately 10 times more than that can be expected to be obtained from marine microbial communities.

Several approaches can be considered to further improve the LOD of our method. One interesting possibility would be the application of external magnetic field, which resulted in a 10-fold improvement in LOD of an SPR-based immunoassay [45]. This is however not possible to implement on our current sensing system. Modifications that can be tested include exploring the effect of: (1) buffer composition used for hybridization and magnetic bead conjugation; (2) magnetic bead size; (3) adding a crowding agent PEG-20000; and (4) testing a competition assay. Eventually, a comparison between similar sized gold-nanoparticles and streptavidin coated magnetic beads may be worth exploring to investigate the potential role of plasmonic coupling in gold-nanoparticle based signal enhancement. Gold-nanoparticle based studies generally report lower LOD. For example, 1 pM of fragmented 16S rRNA was established as LOD with an SPRi system, where a similar sequential signal amplification strategy was used, however with streptavidin coated quantum dots (~15–20 nm in diameter), instead of magnetic beads and an incubation time of 3 h [12]. Interestingly, streptavidin signal amplification alone was successful in reaching a similarly low detection limit (9 pM of a 22-nucleotide long microRNA) in 30 min with the industry standard Biacore X^TM^ instrument [14]. Another gold-nanoparticle (20 nm) based signal amplification SPR assay reached 0.01 ng/mL detection limit for 16S rRNA of pathogenic bacteria [46]. A competition assay, where oligo-conjugated gold nanoparticles competed with the target analyte for the binding sites on the SPR surface reached a similar LOD (0.5 nM) for a 22-nucleotide long target, miRNA-200b as our approach [31]. The reaction time in their study was 1 h in a stationary mode of a four-channel SPR instrument [47]. Lastly, an ultrasensitive surface plasmon resonance enhanced light scattering assay was reported to reach LOD of 2 attomoles (60 fM, 50 µL) of miRNA-122 (22 nucleotide long) using a novel amplification scheme, taking advantage of specific RNA*DNA hybrid-binding antibodies [48].

## 4. Conclusions

The preliminary results reported here are promising for the use of morpholino probe based SPR detection of nucleic acids in monitoring bacterial species, hence oil pollution events based on the occurrence of bacterial indicators such as *Oleispira antarctica* RB-8. The applicability of a simple functionalization method using morpholino probes on sensor chip surfaces was confirmed showing excellent regeneration of the surface over 85 sample injections. Selectivity of the assay was comparable to that typically obtained with DNA oligos and other SPR-based morpholino functionalized sensors. Up to 16-fold signal amplification was achieved using streptavidin coated magnetic beads in a sequential injection manner. Detection limits were on the same order of magnitude (0.5 nM for short oligonucleotides) as those reported for other portable SPR systems, with assay times half of previously reported.

## Figures and Tables

**Table 1:** Sequences of probes and target analyte oligonucleotides

Name	Sequence (5′-3′)	MW (g/mol)
**DP**	CTAGCTAATCTCACTCAGGCTCAT-S-S-amide	8243
**CP**	Bio-TCTACGCATTTCACCGCTACA	6706
**REF**	ATGAGCCTGAGTGAGATTAGCTAG	7457
**PC**	ATGAGCCTGAGTGAGATTAGCTAG/iSp18/TGTAGCGGTGAAATGCGTAGA	14,368
**M1**	ATGAACCTGAGTGAGATTAGCTAG	7441
**M2**	ATGAACCTGAGTTAGATTAGCTAG	7416
**NC**	TGTAGCGGTGAAATGCGTAGA	6550

S-S-amide = disulfide-amide modification. Bio = biotin modification. Mismatched nucleotides in M1 (1 mismatch containing oligo) and M2 (2 mismatch containing oligo) are highlighted in bold and underlined. DP = detection probe, CP = capture probe, REF = reference Oleispira oligo, PC = positive control combined oligo, and NC = negative control (non-complementary oligo).

**Table 2:** Fluidics program set-up showing the duration and flow rate (controlled by the peristaltic pump) of either buffer (during initialization, binding, and flow) or regeneration solution (during regeneration) for each programmed step.

Step	Duration (s)	Pump Speed (µL/min)
Initialization	120	20
Baseline	0	0
Injection	10	0
Flow	240	20
Flush	7	n.a.
Flow	120	20
Regeneration	120	100
Flow	120	100

n.a. = not applicable. The flow rate at the flush step is constant 5 mL/min, regulated by the air pump.

**Table 3:** The extent of signal amplification achieved using 4 different 1× bead amounts (5, 10, 25, or 50 µL) by either the capture or the sequential approach in comparison to no amplification. The amplification factors were calculated by dividing the signal of either sequential or capture enhanced refractive index units (RIU) by that of the RIU of oligo-probe complexes alone. The signal of bead solutions injected alone was subtracted prior to calculating the ratios (*n* = 3).

	Amplification Factors (±SD)			
	5 µL	10 µL	25 µL	50 µL
Capture	1.1 ± 0.03	2.0 ± 0.22	3.5 ± 0.31	6.0 ± 0.6
Sequential	3.2 ± 0.32	5.8 ± 0.38	9.7 ± 3.08	15.5 ± 0.70
